# No Change in Serum Metal Ions Levels After Primary Total Hip Replacement With an Additively Manufactured Dual Mobility Acetabular Construct

**DOI:** 10.1016/j.artd.2022.07.019

**Published:** 2022-08-30

**Authors:** Kyle Alpaugh, Mithun Mishu, Geoffrey Westrich

**Affiliations:** a Adult Reconstruction & Joint Replacement, Hospital for Special Surgery, New York, NY, USA; b Division of Hip & Knee Replacement, Department of Orthopaedic Surgery, Massachusetts General Hospital, Boston, MA, USA

**Keywords:** Modular dual mobility, Serum metal ions

## Abstract

**Background:**

Modular junctions of mixed metals have been associated with fretting and corrosion, and in extreme circumstances, adverse local tissue reactions. Since modular dual mobility (MDM) hip constructs involve a titanium shell with a modular cobalt-chromium liner, the aim of this study was to evaluate serum metal ions at minimum 1 year following total hip arthroplasty (THA) in a cohort of patients with these types of implants.

**Methods:**

A single surgeon enrolled 30 patients in a prospective study in which all patients were evaluated preoperatively with serum cobalt, chromium, and titanium metal ion levels. Patients underwent primary THA with an additively manufactured titanium acetabular shell, MDM cobalt-chromium liner, titanium cementless stem, and ceramic head. A “Four Quadrant Test” was used to ensure proper liner seating intraoperatively. At minimum 1 year following surgery, clinical and radiographic evaluation was conducted, and repeat metal ion levels were collected. Patient-reported outcome measures were collected preoperatively and postoperatively.

**Results:**

Twenty-five patients completed 1-year follow-up. All patients had normal metal ion levels for cobalt (<1 μg/L), chromium (<5 μg/L), and titanium (sensitivity test) preoperatively and postoperatively. Patient-reported outcome measures improved significantly after primary THA: Veterans RAND-12 Physical Component Score (31.05 to 45.02, *P* < .001), Visual Analogue Scale Pain score (70.68 to 7.77, *P* < .001), Hip Disability and Osteoarthritis Outcomes Score, Joint Replacement (51.99 to 86.97, *P* < .001).

**Conclusions:**

No significant elevation was detected in serum metal ion levels 16 months following THA using an additively manufactured titanium acetabular shell, a cobalt-chromium MDM liner, and titanium stem with a ceramic head.

## Introduction

Modular dual mobility (MDM) constructs are being used with increasing frequency, and the American Joint Replacement Registry in 2018 noted that 12% of primary and 30.6% of revision total hip arthroplasties (THA) utilized a dual mobility implant [1]. MDM constructs are comprised of an outer titanium acetabular shell joined to a modular inner cobalt-chromium liner through a taper which is mated intraoperatively. Design features of this bearing surface that favor reduced instability risk are greater impingement-free range of motion and increased jump distance by way of increased head-neck offset and larger effective femoral heads, respectively, [2].

However, modularity between the inner cobalt-chromium liner and outer titanium shell introduces another taper junction for potential fretting and corrosion of a mixed metal interface. Secondary to cases of adverse local tissue reaction (ALTR) as the result of fretting and corrosion at other modular metal-on-metal interfaces in total hip replacement, there are concerns of taper corrosion and elevated metal ions levels after implantation of MDM components [[3], [4], [5]]. Furthermore, although it is quite rare, there have been reports of fretting and corrosion among some MDM liners [[6], [7], [8]].

Contemporary evidence has demonstrated good outcomes following implantation of an MDM liner during primary THA [[9], [10], [11], [12]]. Studies have also reported a low rate of serum metal ion elevation and subsequent revision due to ALTR caused by metal ion release at the modular taper junction of conventional MDM liners [[9], [10], [11], [12], [13]].

To our knowledge, there is no evidence documenting the serum metal ions and subsequent outcomes of patients after undergoing implantation with a thinner, more modern, additively manufactured 3-dimensional titanium shell, cobalt-chromium liner, titanium stem, and ceramic femoral head. The purpose of this study is to prospectively evaluate and compare serum cobalt, chromium, and titanium levels preoperatively and at 1-year minimum follow-up. The secondary aim of the study is to report on the outcomes of this patient cohort.

## Methods

At a tertiary care institution, a single surgeon (G.W.) enrolled 30 patients in a prospective study in which preoperative, prior to their primary THA, serum cobalt, chromium, and titanium metal ion levels of all patients were evaluated. Inclusion criteria included patients with no other orthopedic implants. Indications for an MDM liner included patients who the senior author (G.W.) felt was at greater risk of instability. All patients underwent primary THA through a posterolateral approach with an additively manufactured titanium Trident II acetabular shell (Stryker, Mahwah, NJ), a modular cobalt-chromium liner (Stryker), a Secur-Fit titanium cementless stem (Stryker), and a ceramic head (Biolox; CeramTec Medical, Plochingen, Germany). This study was approved by our institutional review board.

Meticulous attention was paid to ensuring that the inner cobalt-chromium liner was well seated within the outer titanium shell using a “4 Quadrant Test” [14]. In brief, the acetabulum rim is completely cleared of debris to ensure visualization of the acetabular component rim and inner taper. The inner surface is irrigated and dried to remove any remaining debris and confirming that any acetabular screws are not prominent. The inner liner is inserted, ensuring that the locking tabs are lined up. Forceful impaction is utilized to seat the liner within its taper. The liner is then divided into 4 quadrants (superior, inferior, anterior, and posterior). All 4 quadrants are tested with the impactor on the rim of the inner liner. Motion or toggling of the liner in the opposing quadrant is evidence of malseating. Should this occur, the MDM liner should be removed, and the acetabular rim inspected for any interposing soft tissue or osteophytes. Further debridement should be performed as needed, and then the MDM liner should be reinserted, again ensuring that the locking tabs are properly lined up with the recesses in the acetabular shell. After testing all 4 quadrants to confirm a congruent inner liner, a final impaction is made centrally in the liner to ensure that it is fully engaged in the taper. Postoperative radiographs at follow-up were assessed for malseated liner according to Romero et al. [15]. All liners were noted to be well seated without any radiographic evidence of malseating.

After a minimum of 1 year following surgery, patients returned for evaluation and repeat measurement of serum cobalt, chromium, and titanium metal ion levels as well as postoperative radiographs. Postoperative radiographic evaluation consisted of an anteroposterior pelvic radiograph and cross-table lateral to evaluate seating of the MDM liner. Additionally, patient-reported outcome measures (PROMs) were collected preoperatively and at 1 year postoperatively. PROMS evaluated in this study were Visual Analogue Scale Pain, Veterans RAND-12 Physical and Mental Component scores, Hip Disability and Osteoarthritis Outcomes Score, Joint Replacement, (HOOS Jr.), and Harris Hip Scores. Statistical analysis was carried out using Chi-square test for categorical variables. Continuous variables were analyzed with a student’s t-test. Significance was set at *P* < .05.

## Results

Of the 30 patients that were enrolled in the study and had preoperative metal ion levels, 25 (83%) were available to complete the study. Five (17%) patients refused to return for follow-up due to their desire not to travel during the pandemic but were asymptomatic and reported no complications since their surgery. The demographic information is depicted in Table 1. There was no significant difference between the group completing follow-up and the overall cohort. Radiographic review at follow-up demonstrated that none of the MDM liners were malseated.

**Table 1:** Demographics.

Patient demographics	MDM acetabular constructs (n = 25)
Age (y ± SD)	63.68 ± 9.58
Gender	
Male (%)	20 (80%)
Female (%)	5 (20%)
BMI (kg/m^2^ ± SD)	29.83 ± 5.08
Mean follow-up (mo ± SD)	16.76 ± 5.07
Laterality	
Right	12
Left	13

SD, standard deviation.

### Metal ion levels

Serum cobalt, chromium, and titanium levels were undetectable preoperatively and again at an average of 16 months postoperatively (Table 2).

**Table 2:** Metal ion levels for cobalt, chromium, titanium.

Metal	Reference range	Preoperative (n = 25)	Postoperative (n = 25)
Cobalt (μg/L)	Normal, <1.0	<1.0	<1.0
Chromium (μg/L)	Normal, <5.0	<1.1	<1.5
Titanium (ng/L)	Normal, none detected	None detected	None detected

### Outcomes

Postoperatively, there was a significant reduction in pain and improvement in function. There was significant improvement across PROMs (Table 3).

**Table 3:** Patient-reported outcomes measures.

Patient reported outcome measures	Preoperative (±SD)	Postoperative (±SD)	*P* value
VAS Pain	70.20 ± 18.17	7.13 ± 11.16	<.001
VR-12 Physical Component	30.34 ± 9.27	45.20 ± 8.68	<.001
HOOS, Jr.	52.22 ± 13.05	87.44 ± 13.47	<.001
Harris Hip ROM	48.47 ± 14.29	91.19 ± 8.51	<.001

ROM, range of motion; SD, standard deviation; VAS, visual analogue scale; VR-12, veterans RAND-12.

### Complications

There were no intraoperative or 90-day complications reported. During the follow-up period, no dislocation or ALTR has been reported or identified among this cohort.

## Discussion

In this prospective study, we demonstrate that patients undergoing primary THA with a cobalt-chromium MDM liner and an additively manufactured 3-dimensional titanium shell did not have elevated serum metal ions levels at a minimum of 1 year after surgery and at average 16 months, postoperatively. Furthermore, the patients in this group experienced significant pain reduction and improvement in function and did not suffer from dislocation or ALTR during the follow-up period.

The phenomenon of metal ion release from total hip replacements has been well documented [3,4]. Metal ion release can occur at the modular interface of tapered junctions via mechanically assisted crevice corrosion, which is a combination of fretting and crevice corrosion [4,8]. MDM implants introduce an additional junction that can act as a potential source of mechanically assisted crevice corrosion leading to metal ion release [[7], [8]]. Increasing utilization of MDM components in patients at increased risk of postoperative hip instability has spurred studies evaluating serum metal ion levels in this unique patient population.

There have been several prospective studies on the change in serum metal ion levels following implantation of a primary MDM construct using an older nonadditively manufactured titanium acetabular shell (Trident). Markel et al. evaluated 39 patients with a titanium shell, press-fit cobalt-chromium liner, and titanium stem with a 28-mm ceramic femoral head [13]. In that study, 20 patients (51% follow-up) had preoperative and 1-year postoperative serum ion levels. There was no difference in serum ion levels at 1 year and again at 2 years. Nam et al. also reported on serum metal ion levels in adult patients younger than 65 years with a primary MDM construct, titanium femoral stem, and either a 22-mm cobalt-chromium or a 28-mm ceramic femoral head and found no overall difference in mean preoperative and 2-year postoperative levels of serum cobalt, chromium, or titanium [11]. Barlow et al. evaluated 4 bearing surfaces including 20 patients with MDM liners [12]. No significant difference was seen in postoperative serum levels of cobalt, chromium, or titanium in the MDM cohort when compared to metal-on-polyethylene, ceramic-on-polyethylene, and ceramic-on-ceramic surface bearings [12]. Our study corroborates these results by showing that there were was no increase in serum metal ion levels (cobalt, chromium, and titanium) at 16 months, on average, postoperatively with a newer MDM construct.

The influence of femoral head composition in MDM constructs on serum metal ion level has been minimally investigated. In a retrospective review by Matsen et al., metal ion levels were measured as part of surveillance among patients who received an MDM construct at primary THA [16]. They noted elevated serum cobalt levels in a subset of patients, and 2 cases of potential ALTR were identified through advanced imaging. The majority of patients in that study were implanted with a cobalt-chromium femoral head on a titanium stem. This introduces another mixed-metal contact site for potential corrosion and trunnionosis that could result in elevated serum ion levels independent of the MDM interface [3,4]. A separate study comparing bearing surfaces also found serum cobalt ion levels to be higher in patients with MDM constructs and a metal femoral head than in those with MDM constructs with a ceramic head [12]. In a study of primary and revision MDM liners with only ceramic heads, Chalmers et al. did not find an increase in serum metal ion levels [10]. Lastly, a recent systematic review has suggested that the use of a ceramic femoral head in conjunction with an MDM construct may mitigate elevation in serum ion levels [17]. Our study, which analyzed MDM constructs with only ceramic heads, supports the findings of the prior studies. We recommend, when possible, the use of a ceramic head in all MDM constructs.

Malseating or canting of the inner cobalt-chromium liner has also been shown to increase the risk of fretting and corrosion in MDM constructs [15]. However, all the liners in this series were well seated, and there was no change in or elevation of serum metal ions. Intraoperatively, great care was taken to evaluate the inner liner and ensure proper seating with taper engagement. Postoperatively, none of the liners were noted to be malseated on follow-up radiographs. Ensuring that the liner is well seated in its taper junction is of critical importance for long-term durability of this construct, and we recommend utilization of a “4 Quadrant Test” to reduce the occurrence of MDM liner malseating [14].

The current study demonstrates similar findings to prior studies demonstrating improvements in patient pain and function resulting in good to excellent outcomes after undergoing primary THA with an MDM construct [[9], [10], [11], [12], [13]]. Lyman et al. demonstrated that substantial clinical benefit thresholds for HOOS Jr. scores ranged from 18 to 36 points using an anchor-based method [18]. The mean improvement in HOOS Jr. scores in our cohort was 35 points indicating that all the patients in this study experienced substantial clinical benefit after undergoing primary THA with this MDM construct. Although there are immediate benefits of improved function, less pain, and a lower dislocation rate [2,9,19], the long-term effects of metal ions levels with MDM constructs are not fully understood, and we recommend continued surveillance of this patient population [[20], [21]].

### Strengths

The strength of this study was that it is a prospective assessment of a single modern MDM construct with a new additively manufactured acetabular shell in a cohort of patients without any other joint replacements to eliminate any confounding variables and whose serum ion levels have not been prospectively reported in the literature. We also obtained preoperative serum metal ion levels to compare the postoperative results. Although the study population is relatively small, we report a high follow-up rate with a cohort comparable in size to other studies [13]. We also report on PROMs and demonstrate a significant improvement in patient function and pain reduction without complications including normal serum ion levels in patients with a well-seated MDM construct.

### Limitations

The limitations of this study include a small overall sample size when compared to the number of MDM constructs circulating in the general population. A control group and longer-term follow-up are ways to improve the applicability of these data. However, it should be noted that prior studies have demonstrated no significant difference in metal ion levels among differing bearing surfaces when paired with a ceramic head and no significant change in metal ion levels between 1 year and 2 year or longer follow-up [[10], [11], [12], [13],^20^]. Despite this, we provide data on this specific MDM construct with an additively manufactured acetabular shell which are absent from the literature, as well as early results on serum metal ion surveillance with this specific implant.

## Conclusions

There was no significant elevation in serum metal ion levels at minimum 1 year (average 16 months) following THA using an additively manufactured titanium acetabular shell, a cobalt-chromium MDM liner, and titanium stem with a ceramic head. Utilizing the “4 Quadrant Test” intraoperatively to ensure proper MDM liner engagement with the shell, we observed no evidence of liner malseating radiographically. Additionally, postoperative pain and function scores improved significantly after surgery.

## Conflicts of interest

G. Westrich receives royalties from Exactech and Stryker Orthopaedics; is in the speakers' bureau of or gave paid presentations Stryker Orthopaedics and Mallinckrodt Pharmaceuticals; is a paid consultant for Stryker Orthopaedics; receives research support from Stryker Orthopaedics; and is a board member of Eastern Orthopaedic Association. The other 2 authors declare no potential conflicts of interest.

For full disclosure statements refer to https://doi.org/10.1016/j.artd.2022.07.019.

## Appendix A Supplementary data

Conflict of Interest Statement for Alpaugh

Conflict of Interest Statement for Westrich

Conflict of Interest Statement for Mishu
