# Supplementary material for: No Change in Serum Metal Ions Levels After Primary Total Hip Replacement With an Additively Manufactured Dual Mobility Acetabular Construct
Source: Arthroplast Today. 2022 Aug 30;17:132–5. doi: 10.1016/j.artd.2022.07.019 (PMC9449544; doi:10.1016/j.artd.2022.07.019)
Supplement: Conflict of Interest Statement for Westrich [file mmc2.pdf]

INDIVIDUAL CONFLICT OF INTEREST STATEMENT

American Association of Hip and Knee Surgeons

(Adopted from the American Academy of Orthopaedic Surgeons disclosure statement)

The following form **must be filled out completely and submitted by each author (example, 6 authors, 6 forms). All items require a response. If there is no relevant disclosure for a given item, enter "*None*."**

Manuscript Title: No Change in Serum Metal Ions Levels After Primary Total Hip Replacement with an Additively Manufactured Dual Mobility Acetabular Construct

1. Royalties from a company or supplier (The following conflicts were disclosed)
Exactech, Stryker Orthopaedics

2. Speakers bureau/paid presentations for a company or supplier (The following conflicts were disclosed)
Exactech, Stryker Orthopaedics, Mallinckrodt Pharmaceuticals

3A. Paid employee for a company or supplier (The following conflicts were disclosed)
None

3B. Paid consultant for a company or supplier (The following conflicts were disclosed)

Exactech, Stryker Orthopaedics

3C. Unpaid consultants for a company or supplier (The following conflicts were disclosed)

None

4. Stock or stock options in a company or supplier (The following conflicts were disclosed)

None

5. Research support from a company or supplier as a Principal Investigator (The following conflicts were disclosed)

Exactech, Stryker Orthopaedics

6. Other financial or material support from a company or supplier (The following conflicts were disclosed)

None

7. Royalties, financial or material support from publishers (The following conflicts were disclosed)

None

8. Medical/Orthopaedic publications editorial/governing board (The following conflicts were disclosed)

None

9. Board member/committee appointments for a society (The following conflicts were disclosed)

Eastern Orthopaedic Association

Each author must sign AND print or type his/her name, date and submit a separate form

In addition, one BLINDED Conflict of Interest form (no author names used) should be submitted per manuscript with all author disclosures.

Geoffrey Westrich	[signature]	12/01/2021
Author Name (Print or Type)	Author Signature	Date
